# The efficacy of cognitive behavioral therapy for suicidal ideation in depression:a systematic review and network meta-analysis of randomized clinical trials

**DOI:** 10.3389/fpsyt.2025.1675224

**Published:** 2025-11-28

**Authors:** Wenyan Huang, Qun Pan, Hui Ma, Lina Na, Hao Yang, Mengjiao Wu

**Affiliations:** Department of Medical Psychology, The Affiliated Brain Hospital of Nanjing Medical University, Nanjing, China

**Keywords:** depression, cognitive behavioral therapy, suicidal ideation, network meta-analysis, suicide

## Abstract

**Background:**

Suicidal ideation exhibits a strong correlation with mortality rates among individuals diagnosed with depression. Cognitive behavioral therapy (CBT) may exert an influence on suicide-related outcomes in patients with depression. We examined the impact of CBT on suicidal ideation and depressive symptoms in depression.

**Methods:**

By searching PubMed, Cochrane Library, EmBase, Web of Science, and PsycINFO databases (from inception to December 31, 2024), we identified randomized controlled trials (RCTs) of CBT for patients with depression. Suicidality was assessed explicitly as an endpoint utilizing clinically established psychometric scales with validated properties. The trials compared CBT for depression against control conditions in adults with depression, and reported findings pertaining to suicidal ideation. Independent pairs of reviewers were responsible for selecting studies and extracting relevant data. We evaluated the effects of CBT on suicide outcomes through random-effects model pairwise and network meta-analyses utilizing Stata 18.0. Risk of bias was assessed with the Cochrane risk-of-bias tool (version 2).

**Results:**

In this systematic review and network meta-analysis, 12 randomized clinical trials were selected from 14 articles (as 3 articles came from the same study and were therefore analyzed together), involving 1336 participants. In terms of suicidal ideation, after the intervention, CBT demonstrated comparable immediate efficacy in diminishing suicidal thoughts when compared to conventional treatment and the control condition. During follow-up, CBT was more effective than the control group (SMD=-6.98, 95%CI (5.71,8.24)). In terms of depressive symptoms, after the intervention, CBT had certain advantages compared with the control group (SMD = 3.11, P<0.05). During follow-up, CBT was more effective than the control group (SMD = 4.54, 95%CI (2.14, 6.95)).

**Conclusion:**

CBT may reduce suicidal ideation and depressive symptoms among patients with depression. However, more randomized controlled trials (RCTs) are needed to elucidate this effect. In psychological intervention trials for depression, monitoring and reporting of suicide-related adverse events should be enhanced.

**Systematic Review Registration:**

https://www.crd.york.ac.uk/PROSPERO/view/CRD42024626377, identifier (PROPSERO).

## 1 Introduction

Suicide is a major cause of death and disability worldwide. Annually, over 700,000 individuals worldwide die from suicide, ranking as the fourth major cause of mortality among individuals aged 15-29 (1). Suicide can be conceptualized as a continuum, extending from suicidal ideation (SI, the belief that life is not worth living or the contemplation of terminating one’s life) to suicide attempts (acts of self-harm intended to lead to death) to suicide deaths (intentional, self-inflicted fatalities) (2). So both suicidal ideation and attempts need to be taken seriously (3).

Depression is a major risk factor for suicide (4). Approximately 50% of the approximately 800,000 suicides that occur annually worldwide take place during depressive episodes, with those suffering from depression exhibiting a nearly 20-times higher likelihood of dying by suicide than the general populace (5). Statistics indicate that the probability of suicidal ideation among depressed patients ranges from 65% to 80%, with 15% to 25% of patients reporting lifelong suicidal ideation (6), and more than 4% of depressed patients completing suicide (7). According to the World Health Organization (WHO), the suicide rate among individuals with depression is 1 in every 100, and depression serves as the primary diagnosis in approximately 50% of all suicide cases. Suicidal ideation is prevalent among 50% to 75% of depressed patients (8, 9), with approximately 30% of these patients having a history of suicide attempts (10). Researchers have proposed that suicide is the most prevalent symptom of depression, in addition to its core symptoms of low mood and anhedonia (11). Depression emerges as a significant and powerful predictor of suicidal ideation (4). Over an extended period, a significant number of patients struggle to fully recover from depressive episodes. They often experience residual symptoms that substantially impede their psychosocial functioning (12). These residual symptoms are a crucial factor contributing to both suicide risk and relapse (13).

Therefore, identifying interventions capable of effectively decreasing the incidence of depressive symptoms and suicidal events carries considerable public health importance. Pharmacotherapy continues to be the favored approach for the clinical management of depression, as studies have indicated that antidepressants can diminish suicidal thoughts and actions, particularly more effective in mitigating suicidal ideation compared to preventing suicide attempts. However, this finding does not exclude the likelihood that antidepressants may also elevate the risk of suicide in specific patient populations, which may be linked to factors such as patient age and the length of antidepressant administration (14). The presence of suicidality in depressed individuals has been linked to cognitive deficiencies (11, 15), implying that clinical enhancement of cognition through a top-down cognitive processing method, psychotherapy, may decrease suicide risk. Therefore, NICE guidelines the presence of suicidality in depressed individuals has been linked to cognitive deficiencies (11, 15), implying that clinical enhancement of cognition through a top-down cognitive processing method, psychotherapy, may decrease suicide risk. Within the realm of psychotherapies, cognitive behavioral therapy (CBT) is distinguished as one of the interventions with the highest level of empirical efficacy based on evidence (16).

CBT can modify dysfunctional beliefs and behavior by confronting and challenging inaccurate cognitive patterns, thereby eliminating negative emotional and behavioral responses in patients (17). Studies have demonstrated that CBT is effective in alleviating depression severity (18) and lowering the recurrence rate of depressive episodes (19, 20), indicating its substantial benefit for depression (21). The third generation of CBT has evolved to emphasize the contextual and functional aspects of cognition, emotion, and behavior, along with situational and experiential processes of transformation. Numerous intervention trials for patients with depression have been conducted, yet there remains a notable lack of focus on the treatment of suicidal behavior, which is intricately tied to mood disorders.

At present, within the pertinent research domains, existing studies exhibit some limitations. First, the scarcity of research focused on suicidal ideation and suicide risk results in inadequate statistical power, thereby impeding a clear elucidation of the precise impact of psychotherapy for depression on suicidal behaviors (2). Moreover, the suboptimal quality of the included studies, combined with the lack of long-term effect assessments of psychotherapy, significantly limits the depth and scope of the research (2). Second, while existing researches have extensively evaluated the influence of psychological interventions on suicidality among individuals with depression, a significant proportion of these studies have failed to concentrate on specific therapeutic approaches, notably cognitive-behavioral therapy (22). Consequently, when analyzing the specific efficacy of CBT, the data obtained are fragmented and lack precision. Finally, prior studies have predominantly employed the traditional meta-analysis approach, which is limited to providing direct evidence for the comparison of therapeutic outcomes (2, 22). The absence of research on the holistic comparison of multiple therapeutic interventions makes it challenging to establish a comprehensive ranking of therapeutic efficacy.

Network meta-analysis (NMA) offers a novel approach to addressing challenges in prior research, especially when head-to-head studies are scarce (23, 24). Unlike conventional meta-analysis, NMA synthesizes treatment comparison outcomes from both direct and indirect evidence within a unified network, thus surpassing its limitations (25). In clinical research, many small-scale randomized controlled trials (RCTs) are constrained by limited sample sizes, which impede the accurate detection of treatment outcome variations (26). NMA effectively addresses this by aggregating samples from these small RCTs, thereby increasing sample size and enhancing the ability to discern result differences. Importantly, NMA holds a distinct advantage over standard meta-analysis in our study context. While the latter often provides fragmented perspectives, NMA, through indirect network comparisons, reduces bias from study-specific characteristics in head-to-head RCTs—bias that may go undetected in conventional research. Additionally, NMA integrates a larger volume of data, aiding a more comprehensive understanding of the research terrain (24). Crucially, NMA enables the ranking of multiple treatment alternatives by constructing an all-encompassing comparative framework. This framework, incorporating both direct and indirect comparisons, allows for a scientific ranking of intervention effectiveness and the formulation of rational treatment recommendations. Given these advantages, particularly in synthesizing evidence from diverse CBT approaches, the present NMA will comprehensively examine the effects of cognitive behavioral therapy on suicide and depressive symptoms among patients with depression.

## 2 Materials and methods

### 2.1 Search strategy

The databases searched included PubMed, Cochrane Library, Embase, Web of Science, and PsycINFO. The search terms used included “depressive disorder”, “major depressive disorder”, “psychotherapy”, “cognitive behavioral therapy”, and “randomized controlled trial”. The literature search covered publications from the inception of each database up to December 31, 2024.

### 2.2 Inclusion and exclusion criteria for literature

#### 2.2.1 Inclusion criteria

(1) Studies must be randomized controlled trials (RCT); (2) Participants must have a standardized diagnosis of depression; (3) Interventions must include cognitive behavioral therapy; (4) Control measures must be pharmacotherapy, treatment as usual (TAU), enhanced treatment as usual (E-TAU), or waiting list control (WLC); (4) Outcome measures must include scores on suicidality-related scales.

#### 2.2.2 Exclusion criteria

(1) Systematic reviews or meta-analyses. (2) Conference communications or abstracts. (3) Studies where the full text is inaccessible or where data extraction is not feasible. (4) Non-English language literature.

### 2.3 Literature screening and data extraction

Two independent investigators conducted literature screening, data extraction, and cross-verification for accuracy. Any discrepancies encountered were resolved either by discussion among the two investigators or through consultation with a third independent investigator. The process began with the removal of duplicate publications. Following the removal of duplicates, an initial screening was performed based on the titles and abstracts to exclude studies that did not meet the predefined inclusion criteria. A subsequent, more detailed screening was then conducted, involving the retrieval and reading of full-text articles to ascertain their final eligibility for inclusion in the analysis.

A custom-designed information extraction table was utilized to gather pertinent data, including (1) the first author’s name, the year of publication, and the title of the study; (2) the diagnostic criteria adopted, the sample size, baseline characteristics of the participants, assessment tools utilized, the intervention and control measures implemented, the duration of the treatment regimen, and the follow-up duration for the research subjects; (3) the outcome indicators and the associated data collected, etc.

### 2.4 Risk of bias evaluation of included studies

The risk of bias in the included studies was assessed using the Cochrane Risk-of-bias tool (version 2). We assessed the risk of bias according to six components: randomisation, deviations from the intended interventions, completeness of outcome data, accuracy of outcome measurements, potential for selection of reported results, and any other potential biases.

### 2.5 Statistical analysis

A network meta-analysis was conducted using Stata SE (version 18.0). The outcome measures were continuous variables. Given the inclusion of outcome indicators that varied in scale, we utilized the standardized mean difference (SMD) as a method to summarize the effect sizes of the variables. The findings reported encompassed both the point estimate and a 95% confidence interval. These values lack units and do not possess inherent significance, serving merely as indicators of the effect’s magnitude.

Firstly, a network plot of evidence for effectiveness was used to assess the appropriateness of adopting the network meta-analysis approach. Secondly, in the presence of closed loops within the network evidence map, we performed tests for consistency and heterogeneity. A two-sided P-value > 0.05 or a 95% confidence interval (CI) encompassing 0 would indicate the absence of significant inconsistency among various interventions. In the absence of a direct link between two interventions, we would resort to an indirect comparison via network meta-analysis. Thirdly, we applied Bayesian random-effects models to compare interventions in pairs. A two-sided P-value < 0.05 or a 95% CI excluding 0 signifies a statistically significant difference.Lastly, we constructed a hierarchy of treatments to compare the relative effectiveness of various interventions based on their surface under the cumulative ranking (SUCRA) values and league table rankings, with treatment as usual as the reference. When a treatment is definitively identified as the least effective option, its SUCRA value will be 0; conversely, when a treatment is unequivocally deemed the most effective, its SUCRA value will be 1.

In this NMA, we merged pharmacotherapy, waiting list control (WLC), and treatment as usual (TAU)/enhanced TAU (E-TAU) into a single “control group”. This is well-justified. These controls offer a common reference for evaluating experimental psychotherapies (MBCT, CBASP, F-CBT, Traditional CBT), enabling a comprehensive relative efficacy assessment. Each control type has inherent variability, like diverse antidepressants in pharmacotherapy. Combining them reduces this impact, focusing on general comparison. It also boosts sample size, enhancing statistical power for precise effect detection. Moreover, it reflects real-world depression treatment scenarios, improving result generalizability for clinical use. Additionally, some control types, like WLC and TAU, are reported in only a limited number of trials, reducing their individual reference value. Hence, merging them into a control group sseems more appropriate.

## 3 Results

### 3.1 Literature search results

**Figure 1:**
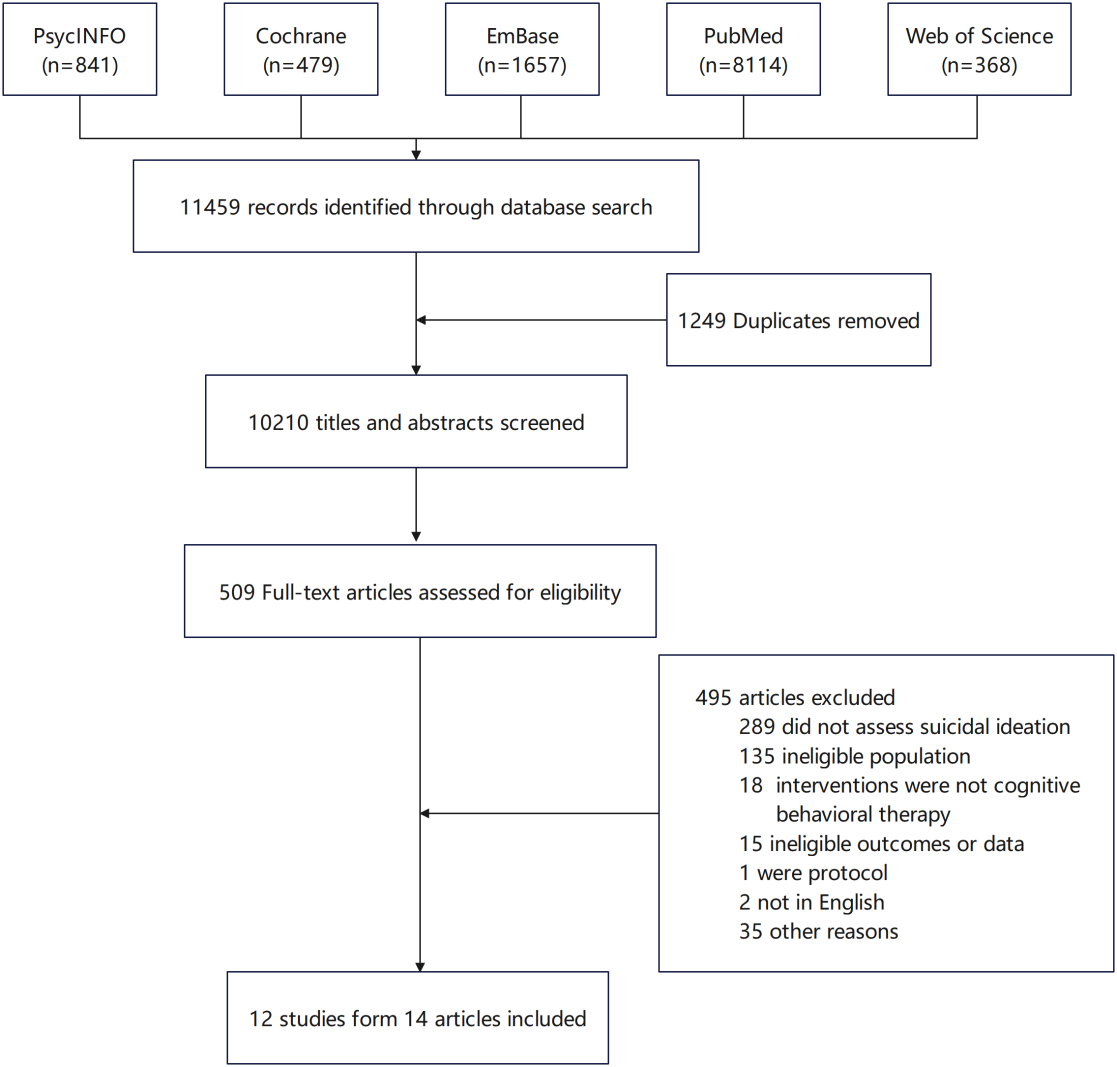
Study selection.

According to the search strategy, a total of 14 articles (including 12 studies, as 3 articles came from the same study and were therefore analyzed together) were ultimately identified. Among the identified articles, there were 7 two-arm studies, 3 three-arm studies, and 2 four-arm studies. The process involving the inclusion and exclusion of studies during the literature search is depicted in **Figure 1**.

### 3.2 Basic characteristics of the included studies

The 12 included studies, involving 1336 participants, including both patients in the acute phase of depression and patients in remission. The sample was broad, ranging from individuals aged 12 to 70 years who had depression. The mean age is 25.23. Most trials reported that there were more female than male participants. However, the ratio of females to males exhibited significant variability both within individual trials (across arms) and among different trials, ranging from very minor differences to a scenario where the number of females was twice that of males. In one trial, the ratio of females to males was similar. Another trial had more male participants than female participants (the participants were veterans). Additionally, two trials did not provide information regarding the gender of the participants.

Among the 12 studies, 6 were published from 2010 to 2020, 4 were published from 2000 to 2010, and the other 2 were published in the 1980s and 2021 respectively. Among these, 6 projects are conducted in the United States, while one project is undertaken in each of the following countries: Australia, the United Kingdom, Switzerland, the Netherlands, Germany, China, India, and Malaysia. Nine trials evaluated suicide-related factors using specific suicide-related scales, whereas three trials assessed these factors utilizing suicide items from the depression scale.

Among the 12 studies, all participants were randomly assigned to different group. The intervention group used cognitive behavioral therapy, 8 trials were based on conventional cognitive behavioral therapy, 3 trials were based on a positive thinking approach, and 1 was based on family-based cognitive behavioral therapy, of which 1 study also used CBASP for comparison. The control group received conventional treatment (11 trials), a placebo (2 trials), and a waiting list (1 trial). Refer to **Table 1** for details.

**Table 1:** Characteristics of included trials in which suicidal ideation was assessed as an outcome.

	Age mean ± SD (years)	Sample size		Treatment		Suicidal ideation assessment tool	Scale for suicidal ideation score					
		Intervention	Control	Intervention	Control		Baseline mean ± SD		End of intervention mean ± SD		Follow-up visits	
							Intervention	Control	Intervention	Control	Intervention	Control
March, et al, 2009 (27)	14.6 ± 1.5	111	109	CBT	Pharmacotherapy	SIQ-Jr	15 ± 0.85	17 ± 0.79	9.5 ± 9.1	12.1 ± 11.1	8.2 ± 8.1	10.5 ± 10.4
Becker-Weidman, 2010 (28)	14.6 ± 1.5	111	221	CBT	Pharmacotherapy	SIQ-Jr	21.8 ± 21	21.8 ± 19.1	11.9 ± 14.9	14.8 ± 17.3		
Sinniah, et al, 2017 (29)	43.13	33	36	CBT	TAU	BSS	28 ± 3.91	28.66 ± 5.06	24.06 ± 5.01	28.42 ± 5.03	24.06 ± 3.51	26.61 ± 7.56
Barnhofer, et al, 2009 (30)	41.93 ± 10.13	16	15	MBCT	TAU	BSS	2.21 ± 2.45	2.78 ± 2.08	1.14 ± 1.79	2.42 ± 2.53		
Esposito-Smythers, et al, 2019 (31)	14.90 ± 1.51	74	73	F-CBT	E-TAU	SIQ-Jr	46.6 ± 22.7	48.8 ± 20.9	16.8 ± 14.2	16.4 ± 14.9	18.6 ± 12.5	16.2 ± 8.1
Forkmann, et al,2014 (32)	43.89 ± 10.02	64	66	MBCT	waiting list	The corresponding items in the Dutch version of the Depression Symptom Scale self-rating table	0.31 ± 0.59	0.20 ± 0.50	0.11 ± 0.36	0.25 ± 0.61		
Forkmann, et al,2016 (33)	50.84 ± 11.92	71	35	MBCT,CBASP	TAU	Hamilton Depression Rating Scale suicide program	0.83 ± 0.81 (MBCT) 1.03 ± 0.89 (CBASP)	0.89 ± 0.90	0.58 ± 0.84 (MBCT) 0.57 ± 0.92 (CBASP)	1.06 ± 1.00		
Weitz, et al, 2014 (34)	35.0	32	76	CBT	Pharmacotherapy	Hamilton Depression Rating Scale suicide program	1.97 ± 0.84	1.92 ± 0.76	0.97 ± 1.18	0.76 ± 1.09		
Melvin, et al, 2006 (35)	15.3 ± 1.5	22	26	CBT	Pharmacotherapy	SIQ-Jr	26.05 ± 19.93	29.42 ± 27.24	19.41 ± 19.64	24.23 ± 26.9	13.5 ± 9.09	20.96 ± 26.12
Miller, et al, 1989 (36)	36.09 ± 13.66	28	17	CBT	TAU	SSI	5.9 ± 10.1	12.5 ± 14.8	8.4 ± 13.1	5.2 ± 11.3	5.1 ± 4.6	6.5 ± 7.8
Shu, et al, 2021 (37)	22.69 ± 3.52	25	21	CBT	Pharmacotherapy	SSI	43.2 ± 9.8	42.5 ± 7.2	8.5 ± 8.6	16.6 ± 13.8		
Pigeon, et al, 2019 (38)	55.02 ± 16.33	24	30	CBT	TAU	C-SSRS-SI	13.04 ± 3.57	12 ± 2.59	6.14 ± 6.76	7.83 ± 6.36		

CBT is cognitive behavioural therapy; MBCT is Mindfulness-based cognitive therapy; F-CBT is Family-focused cognitive behavioral therapy; CBASP is cognitive behavioural analysis system of psychotherapy; TAU is treatment as usual; E-TAU is enhanced treatment as usual; The SIQ-Jr is the Suicidal Ideation Questionnaire - junior high; BSS is the Baker Scale of Suicidal Ideation; SSI is the Suicidal Ideation Scale.

### 3.3 Results of risk of bias evaluation

**Figure 2:**
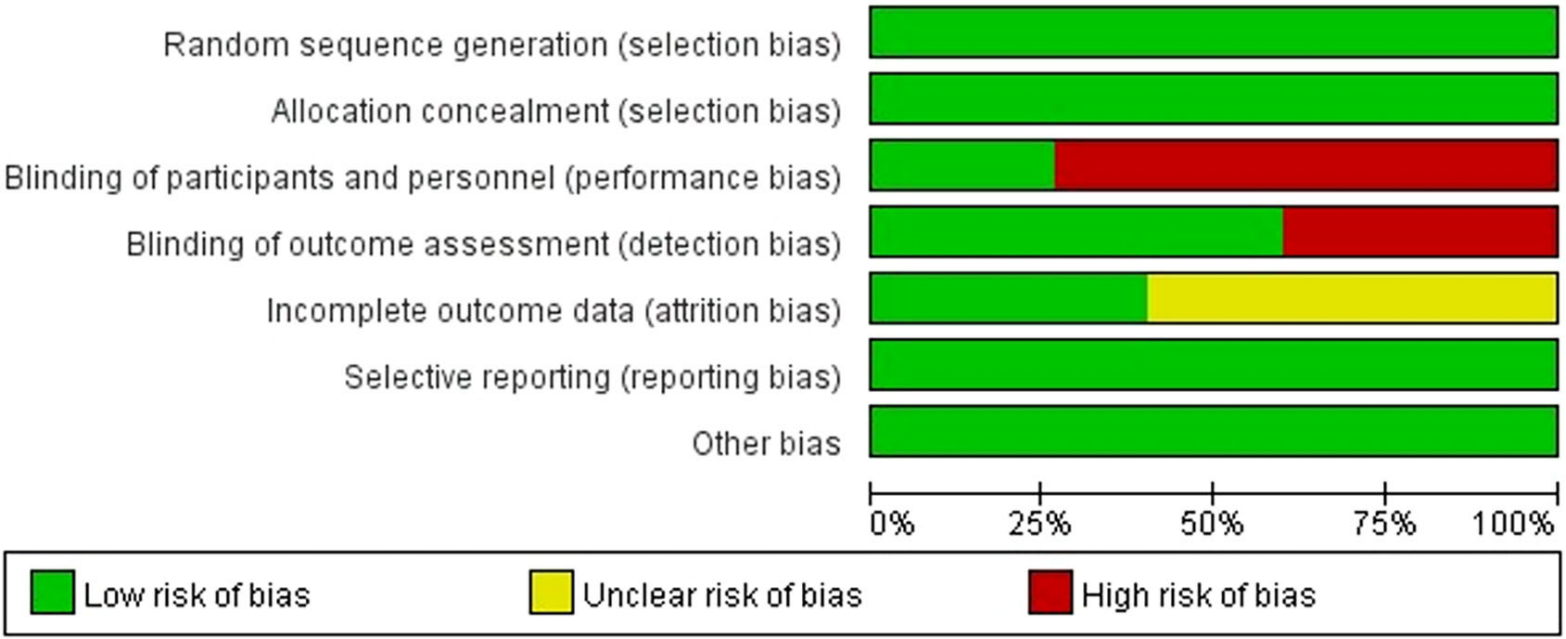
Risk of bias graph of the included studies.

The results of the risk of bias evaluation of the included studies are shown in **Figure 2**. All studies were RCTs and were evaluated as low risk. 3 studies blinded both outcome assessors and patients, 8 studies blinded investigators and were evaluated as low risk, and the studies with inadequate blinding descriptions involved multiple aspects of bias, so the other studies were classified as high risk. 5 studies described adequate information on attrition and were assessed as low risk; the others were not mentioned and were evaluated as uncertain risk. Data from the included literature were complete.

Funnel plots were not used to evaluate publication bias because this method is not sufficiently probative in meta-analyses of a small number of trials with similar sample sizes (39).

### 3.4 Net meta-analysis results

#### 3.4.1 Efficacy of CBT for depressive symptoms

##### 3.4.1.1 End of intervention

**Figure 3:**
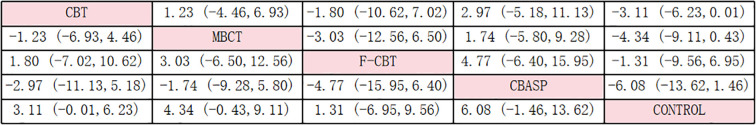
League table of depressive symptoms at the end of the intervention.

In terms of the effectiveness of the intervention for depressive symptoms (**Figure 3**), there was an advantage of CBT over CONTROL (SMD = 3.11, 95% CI (-0.01, 6.23)), but the confidence intervals were relatively wide, suggesting that there is some uncertainty about the exact extent of this advantage.

**Figure 4:**
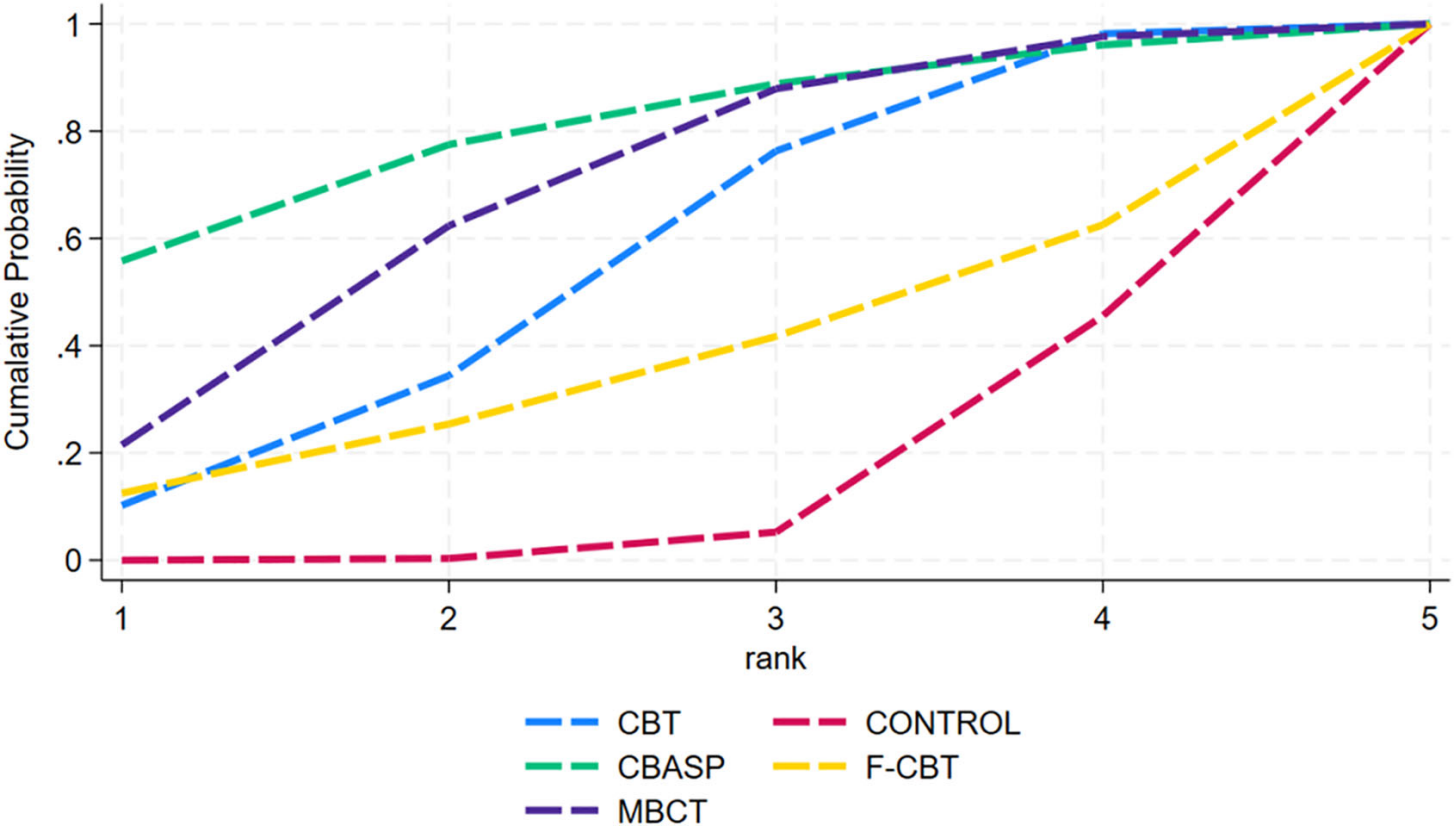
Ranking of cumulative probability of depressive symptoms at the end of the intervention.

The ranked results (**Figure 4**) showed that CBASP was the most effective intervention for depressive symptoms in patients with depression (SUCRA = 79.6), followed by MBCT (SUCRA = 67.3), CBT (SUCRA = 54.8), F-CBT (SUCRA = 35.5), and CONTROL (SUCRA = 12.8), respectively.

##### 3.4.1.2 Follow-up visits

**Figure 5:**
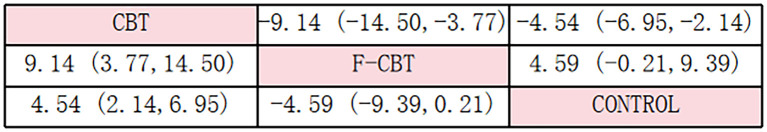
League table of depressive symptoms at the follow-up stage.

5 studies conducted follow-up studies for depressive symptoms. The results of the studies (**Figure 5**) all showed that CBT was more efficacious than CONTROL (SMD = 4.54, 95% CI (2.14, 6.95)) and F-CBT (SMD = 9.14, 95% CI (3.77, 14.50)), and the difference in efficacy between CONTROL and F-CBT was not significant.

#### 3.4.2 Efficacy of CBT on suicidal ideation

##### 3.4.2.1 End of intervention

**Figure 6:**
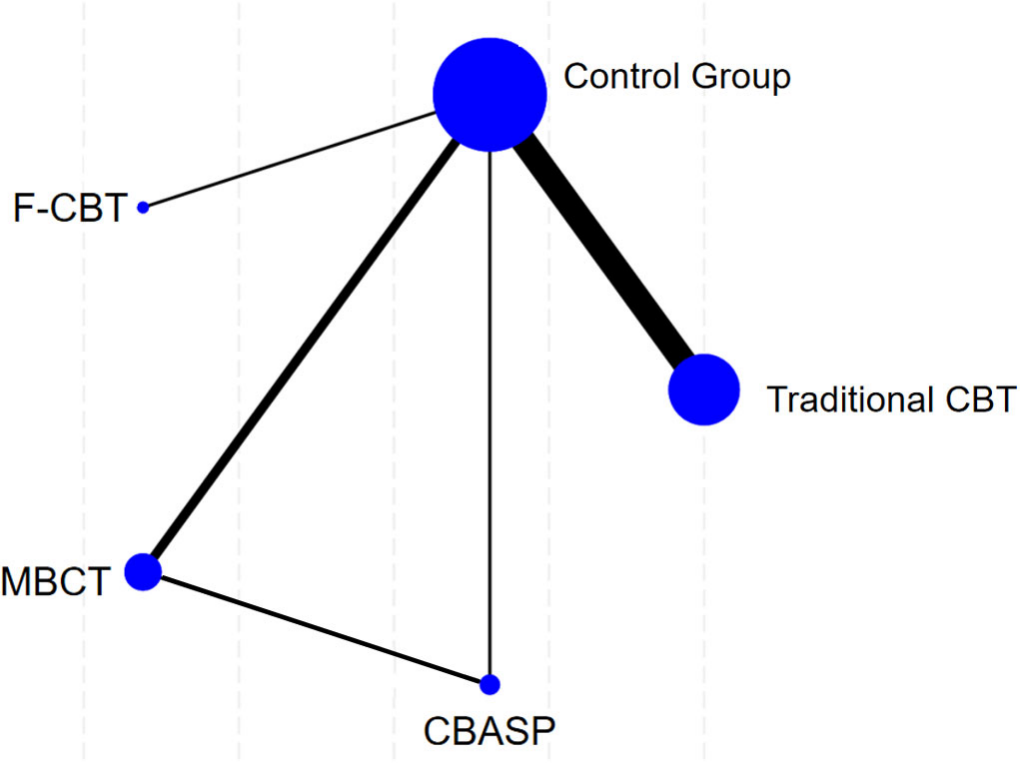
Network plot of therapies comparisons for suicidal ideation for depression. The control group includes Pharmacotherapy and waiting list.

The network diagram is shown in **Figure 6**. The dots in the figure represent different intervention methods, with larger dots indicating larger sample sizes receiving such interventions; thicker connecting lines in the diagram indicate more randomised controlled studies.

**Figure 6** shows the presence of closed loops, so inconsistency tests were performed. The results of the node-splitting method showed that there was no statistically significant difference in inconsistency (p > 0.05). Suggesting good consistency, the study data were analysed using the consistency model.

**Figure 7:**
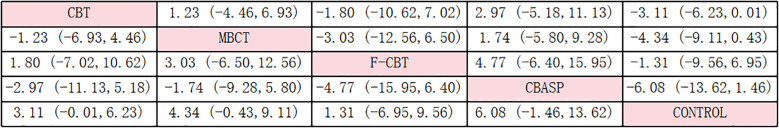
League table of suicidal ideation at the end of the intervention.

No significant differences between therapies were found in the effectiveness of psychotherapy for suicidal ideation (**Figure 7**).

**Figure 8:**
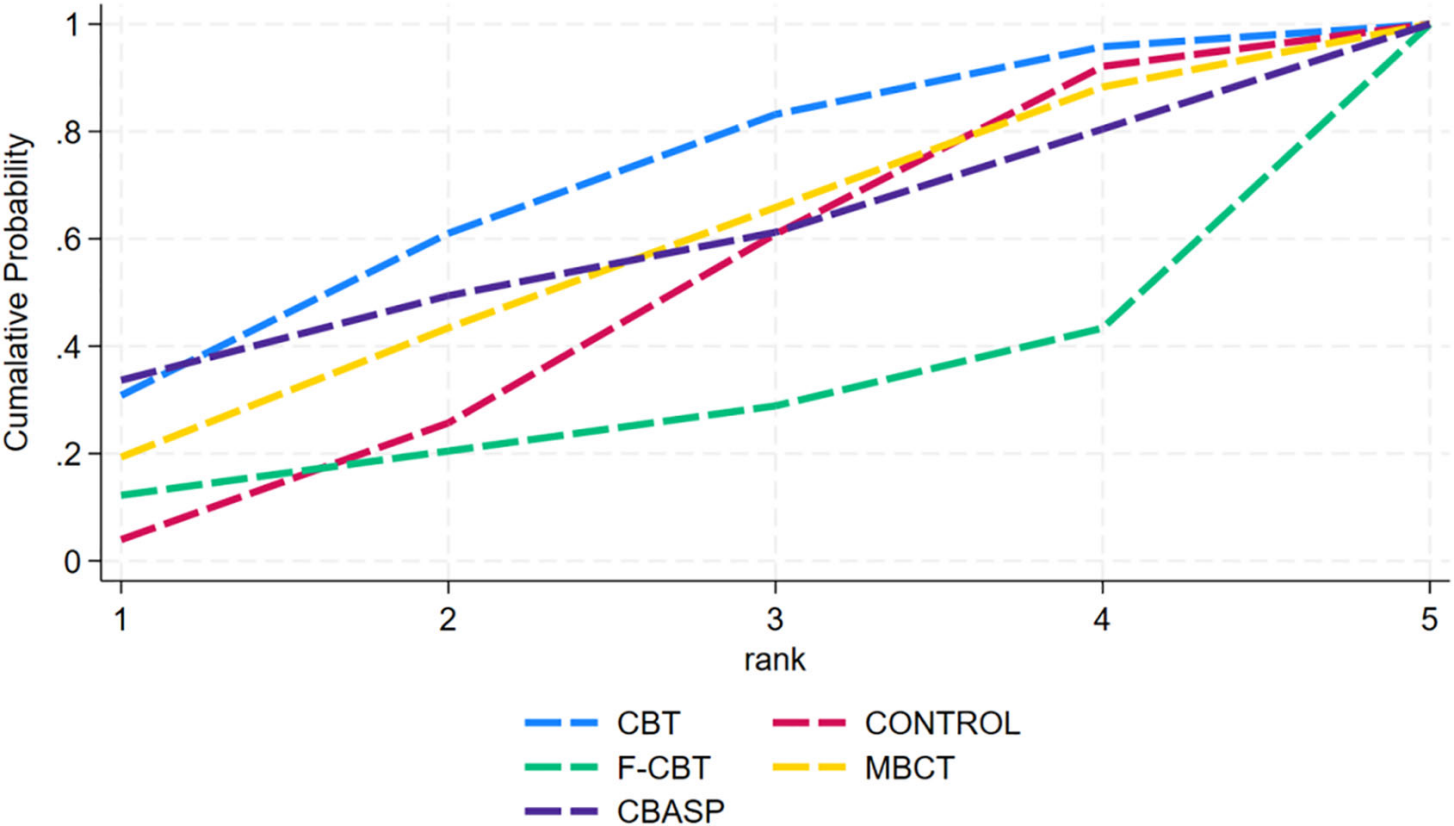
Ranking of cumulative probability of suicidal ideation after the intervention.

The cumulative probability ranking graph was derived from the SUCRA ranking (**Figure 8**). The ranking results showed that CBT was the most effective intervention for suicidal ideation (SUCRA = 67.7), followed by CBASP (SUCRA = 56.2), MBCT (SUCRA = 54.2), CONTROL (SUCRA = 45.7), and F-CBT (SUCRA = 26.2), respectively.

##### 3.4.2.2 Follow-up visits

**Figure 9:**
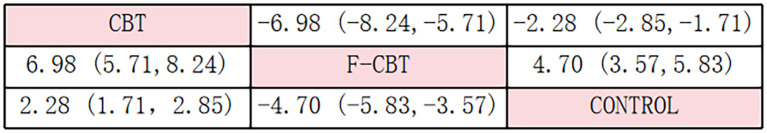
League table for suicidal ideation at the follow-up stage.

**Figure 10:**
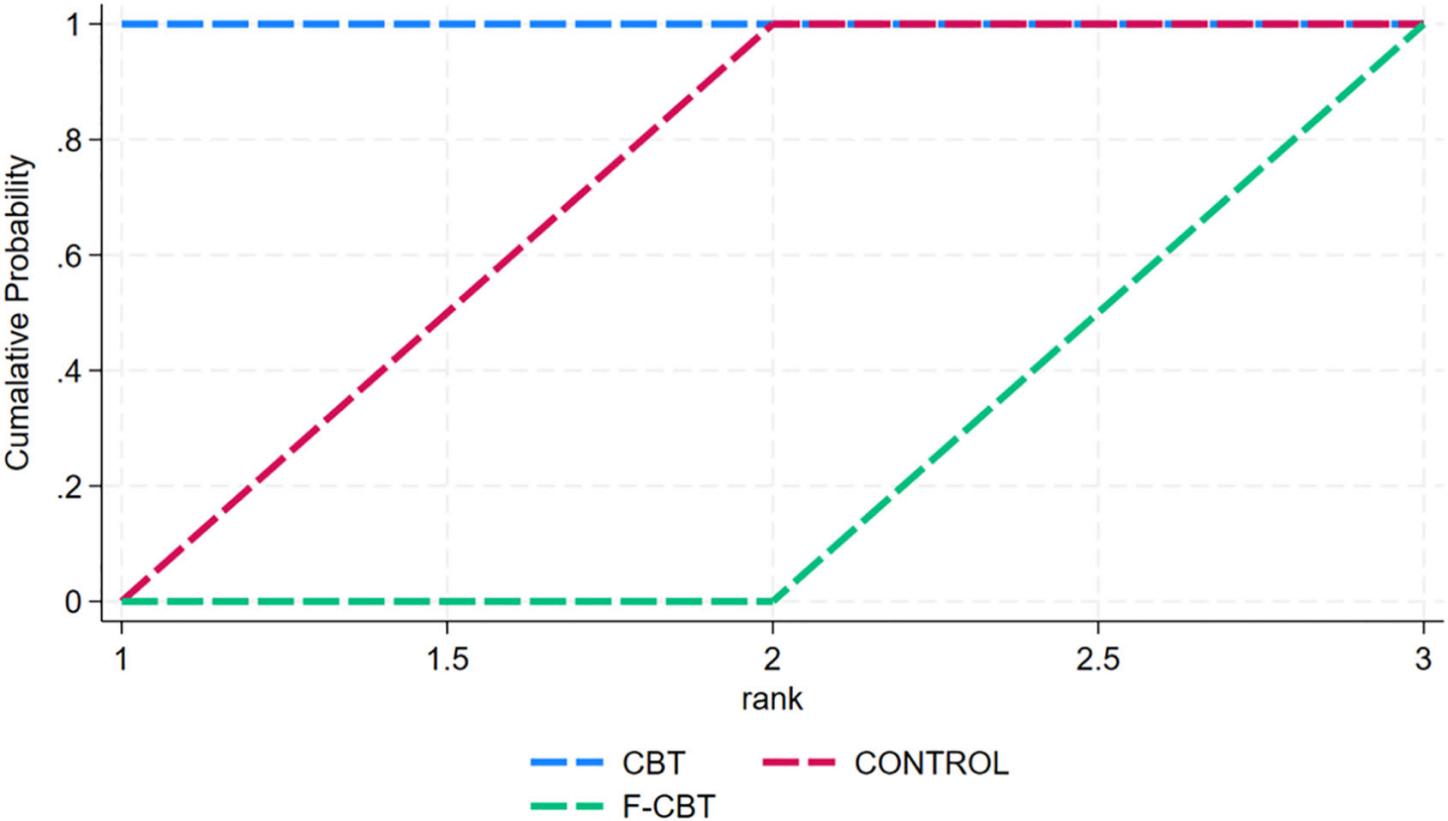
Cumulative probability ranking of suicidal ideation at the follow-up stage.

5 studies conducted follow-up studies for suicidal ideation are shown in **Table 2**. The results of the studies (**Figures 9**, **10**) all showed superior efficacy of both CBT (SMD=-6.98, 95% CI (5.71, 8.24)) and F-CBT (SMD = 6.98, 95% CI (5.71, 8.24)) in the control group and of Pharmacotherapy (SMD = 6.98, 95% CI (5.71, 8.24)) in the F-CBT (SMD = 4.70, 95% CI (3.57, 5.83)).

**Table 2:** Characteristics of included trials assessing suicidal ideation as an outcome during the follow-up stage.

	Follow-up time (months)	Mean age (years) (SD)	Sample size		Treatment	
			Intervention	Control	Intervention	Control
Sinniah, et al, 2017 (29)	6	43.1	33	36	CBT	TAU
Melvin, et al, 2006 (35)	6	15.3 (1.5)	22	26	CBT	Pharmacotherapy
Miller, et al, 1989 (36)	12	35 (13.66)	28	17	CBT	TAU
March, et al,2009 (27)	12	14.6 (1.5)	111	109	CBT	Pharmacotherapy
Esposito-Smythers, et al, 2019 (31)	18	14.9 (1.51)	74	73	F-CBT	E-TAU

CBT is cognitive behavioural therapy; F-CBT is Family-focused cognitive behavioral therapy; TAU is treatment as usual; E-TAU is enhanced treatment as usual.

The cumulative probability ranking graph was derived from the SUCRA ranking (**Figure 10**). The ranking results showed that CBT was the most effective intervention for suicidal ideation (SUCRA = 100.0), followed by CONTROL (SUCRA = 50.0), and F-CBT (SUCRA = 0.0), respectively.

## 4 Discussion

The Sorting of Cumulative Probabilities (SUCRA) visualizes the rankings and compares the effectiveness of different interventions, but does not represent statistically significant differences between specific treatments. Combined with the league table and cumulative probability ranking, our analysis suggests a potential trend indicating that psychotherapeutic approaches (including CBT, MBCT, F-CBT, and CBASP) may demonstrate superior efficacy compared to control conditions. However, it is critical to note that the control conditions in included studies encompassed not only medication monotherapy but also treatment-as-usual (TAU), enhanced treatment-as-usual (E-TAU), and waitlist controls.

After the intervention, both the CBT and control groups exhibited similar immediate effects in reducing suicidal ideation. This similarity may be attributed to the effectiveness of both approaches in alleviating depressive symptoms, which is a critical factor in mitigating suicidality. At this stage, whether through cognitive enhancement (the core component of CBT) (17) or by modulating neurotransmitter levels (the mechanism of action of pharmacological treatments) (5), both methods can effectively relieve the patient’s emotional distress and decrease the risk of suicide in a short term. However, during the follow-up, CBT demonstrated more enduring positive effects on suicidal ideation. This difference may arise from CBT’s dual focus on not only symptom relief but also on equipping patients with positive coping strategies, altering maladaptive thinking patterns, enhancing self-regulation capabilities, and fostering the recovery of psychosocial functions. These skills are particularly vital when patients encounter life challenges and can more effectively prevent the recurrence of suicidal ideation. In contrast, pharmacological treatments may necessitate ongoing use to sustain their efficacy and pose a risk of relapse upon discontinuation.

Only 2 studies included suicide-specific interventions (30, 33), which may be a significant reason for the lack of significant difference in the intervention effect on suicidal ideation between the CBT group and the control group. Several studies have suggested that interventions targeting suicidality should be intensified (29, 30, 32, 40). Firstly, psychological education regarding the relationship between suicidal ideation, suicidal imagery, and suicidal behavior should be enhanced (29, 30, 32). Suicidal ideation and imagery typically arise rapidly and exert an immediate impact (40). Research indicates that suicidal patients often experience intrusive suicidal mental images during their most desperate moments, with the frequency and authenticity of these images significantly correlating with the severity of suicidal ideation (40). Therefore, interventions addressing both suicidal ideation and imagery are essential for mitigating suicide risk. Initially, patients may struggle to comprehend the connection between suicidal thoughts and behaviors. However, as the intervention progresses, they can gradually recognize the link between cognition and behavior and learn how reorganizing their thoughts can positively influence their actions (29). In addition, systematic cognitive training focused on suicidality is necessary to assist patients in altering unhealthy thinking patterns stemming from maladaptive cognition. Only 2 studies employed versions of CBT that incorporate modifications specifically tailored for suicide. Consequently, the integration of exercises that directly and explicitly address suicidal reactions may enhance CBT’s effectiveness in reducing suicide risk among patients with depression.

CBT was shown to be comparable or potentially superior effects to pharmacological treatments in reducing depressive symptoms both at post-intervention and follow-up assessments. However, it is critical to emphasize that the comparator groups in included studies were heterogeneous, encompassing medication monotherapy, treatment-as-usual (TAU) and waitlist controls. This may be because CBT works by directly targeting patients’ negative automatic thoughts, cognitive distortions, and dysfunctional beliefs, fundamentally changing the patient’s cognitive structure and emotional response patterns. The long-term effects of this treatment modality remain significant as it helps patients develop the ability to self-manage and regulate their emotions. Although medicines can quickly relieve symptoms, their effects are often limited, and there are problems such as individual differences, side effects, and recurrence after drug withdrawal. In contrast, CBT provides a more comprehensive and lasting solution designed to help patients fundamentally understand and change their emotional experiences and behavioral patterns.

At the end of the intervention, CBT showed more effective in improving depressive symptoms than the control group, but the comparison of effects on suicidal ideation was not outstanding. This is consistent with results of other clinical reports (41–43). Interventions that can improve depressive symptoms may not necessarily reduce suicidallity. Improving suicidal ideation is more difficult than improving depressive symptoms. This may indicate that the improvement of suicidal ideation is independent of the improvement of depressive symptoms. Some studies have proposed that suicidal ideation is an independent psychological phenomenon or a separate disease entity independent of depressive symptom (41–43). This means that treatments aimed at reducing depression may not necessarily also affect suicidal ideation. Moreover, there is a certain lag in the improvement of suicide compared with depressive symptoms.

Only 2 studies in this review included interventions targeting suicide (30, 33). However, results from long-term follow-up suggest that suicide risk is also reduced after receiving CBT that only targets depressive symptoms. Therefore, CBT targeting depressive symptoms can improve core beliefs and improve suicidal ideation to a certain extent, thereby effectively reducing suicidal ideation. The maladaptive thinking patterns of patients are easily activated by minor triggers, even if it is just a subtle change in mood (44). As patients with depression repeatedly engage in negative thinking, the association between negative emotions and the thought patterns associated with them develops and strengthens. That is, in patients with a history of suicidal depression, changes in mood can reactivate suicidal processing patterns. Rudd introduced the ‘suicidal mode’, which refers to a cognitive-emotional-behavioral network that, when activated, directs synchronous processes that manifest on many different levels, including suicidal ideation, negative affect, physiological arousal, and motivation for suicidal behavior (45). According to Rudd, the cognitive components of the suicide mode are characterized by three core unlovability, helplessness, and poor distress tolerance. The activation of these beliefs, and the ensuing perceptions of helplessness, inadequacy, and incompetence, are thought to increase the risk of suicidal ideation and behavior. Recent researches have shown that cognitions associated with these three types of core beliefs predict suicidal ideation (46). Through the intervention role of CBT, the cognitive appraisal system of patients with depression can be reconstructed to correct core beliefs and suicidal cognitions, and ultimately reduce suicidal ideation.

The CBT group demonstrated significant advantages over the control group in terms of improvements in suicidal ideation and depressive symptoms. However, it is worth noting that the result of one study is far from this perspective. Researchers did not find a difference between the CBT group and the control group in terms of suicidal ideation, and reported that the control group was superior to the CBT group in terms of depressive symptoms.This finding deserves further exploration. After 12 months of continuous treatment, patients’ depressive symptoms got improved, but increased between 12 and 18 months in the F-CBT group and not in the E-TAU group. However, the mean values of depressive symptoms were below the range of clinical significance in all groups. Nonetheless, one reason for this may be that most of the adolescents in the E-TAU group (64%) received treatment before receiving outpatient treatment. Data on the type of outpatient treatment method received were not available, so the possibility that adolescents in the E-TAU had received some form of CBT cannot be ruled out. Even though there were no significant differences between patients in the F-CBT and E-TAU groups at baseline, it is possible that the previous treatment received had a profound effect later in the course of treatment. Another potential reason is that at 12 months after treatment, the study protocol required that all F-CBT participants who were still receiving treatment were terminated from the trial and referred to a community therapist. This research procedure may be useful when single-symptom, low-sensitivity patients are the focus of a treatment trial. However, for patients with more severe mental dysfunction, this approach is mismatched with chronic symptoms or the potential medically induced effects of changing therapists at 12 months.

In summary, CBT demonstrated significant benefits in reducing suicidal ideation and alleviating depressive symptoms, especially in long-term follow-up. This emphasizes the importance of psychological interventions in the treatment of depression and the value of using CBT as a preferred or adjunctive treatment. It also suggests that future researches should further explore the addition of suicide-specific interventions to CBT to reduce suicidality and provide more personalized and comprehensive treatment options for patients with depression.

While NMA excels in statistically ranking treatment efficacy, its results can be misleading, as rank differences may be minor or clinically insignificant, rankings may be biased by meta-analytic biases, and probabilities may be unstable in unsound networks. Given these technical and theoretical limitations, including multiple statistical assumptions and challenges related to intransitivity and inconsistency, NMA results should be interpreted with caution (47).

The review drew the above conclusions, but there are several important limitations and challenges of the included studies, and these also point the way for future research. First, only 14 studies with small sample sizes met the inclusion criteria, resulting in limited statistical power. Second, the large age span and different mental or physical illnesses comorbidity among the study subjects resulted in a large heterogeneity of the sample, which may have affected the accuracy and generalisability of the finding. Therefore, future studies should pay more attention to the homogeneity of the study subjects, which can be achieved by setting stricter inclusion and exclusion criteria in to reduce the heterogeneity between samples. Third, inconsistencies in assessment tools and follow-up assessment times are also important issues facing current research. A large-scale meta-analysis demonstrated moderate concordance between single-item suicidal risk assessments and multi-dimensional measurement tools (48). However, even when reported as continuous statistics (e.g., means ± standard deviations), the aggregation of such ordinal data with truly continuous outcomes introduces statistical heterogeneity and potential measurement bias. To overcome this limitation, future studies should adopt more uniform and standardized assessment tools and set clear follow-up times and assessment nodes to ensure comparable and reproducible results. Finally, this study focused solely on adults, excluding other critical demographic groups, particularly children and adolescents, from the investigation. As a result, a comprehensive evaluation of CBT’s efficacy in preventing suicide among individuals with depression across all age groups is not feasible. Additionally, determining the therapy’s applicability and effectiveness variations across different age groups remains challenging. Future research should prioritize expanding the research sample and conducting an in-depth analysis of CBT’s intervention mechanisms and therapeutic outcomes. These efforts will refine the research paradigm for depression suicide prevention and enable the development of more personalized and effective treatment strategies for patients of all ages. In conclusion, future studies should make improvements in terms of reducing sample heterogeneity, harmonizing assessment tools and follow-up time, to obtain more accurate, reliable and generalizable research results and provide stronger support for the development of related fields.

## Data Availability

The original contributions presented in the study are included in the article/supplementary material. Further inquiries can be directed to the corresponding authors.
