# Supplementary material for: The efficacy of cognitive behavioral therapy for suicidal ideation in depression:a systematic review and network meta-analysis of randomized clinical trials
Source: Front Psychiatry. 2025 Nov 28;16:1675224. doi: 10.3389/fpsyt.2025.1675224 (PMC12698522; doi:10.3389/fpsyt.2025.1675224)
Supplement: Supplementary file 1 [file Table1.docx]

Appendix

Search strategy

Search strings for four bibliographical databases.

PubMed

#1

Psychotherapy [Mesh Terms] OR psychotherap*[All Fields] OR psychotherapy [All Fields] OR psychotherapies [All Fields] OR psychotherapeutic [All Fields] OR psychotherapeutics [All Fields] OR psychotherapeutical [All Fields] OR cbt [All Fields] OR 'cognitive behavioural therapy' [All Fields] OR 'cognitive behavioural therapies' [All Fields] OR 'cognitive behavioral therapy' [All Fields] OR 'cognitive behavioral therapies' [All Fields] OR 'Cognitive Behav* therap*' [All Fields] OR 'behav* therap*' [All Fields] OR 'behavior therapy' [All Fields] OR 'behavior therapies' [All Fields] OR 'behavior therapeutic' [All Fields] OR 'behavior therapeutical' [All Fields] OR 'behavior therapeutics' [All Fields] OR 'behavior therapeutist' [All Fields] OR 'behavior therapeutists' [All Fields] OR 'behavior treatment' [All Fields] OR 'behavior treatments' [All Fields] OR 'behaviors therapies' [All Fields] OR 'behaviors therapy' [All Fields] OR 'behaviors therapeutics' [All Fields] OR 'behaviors therapeutic' [All Fields] OR 'behaviors therapeutical' [All Fields] OR 'behaviors therapeutist' [All Fields] OR 'behaviors therapeutists' [All Fields] OR 'behaviors treatment' [All Fields] OR 'behaviors treatments' [All Fields] OR 'behavioral therapies' [All Fields] OR 'behavioral therapy' [All Fields] OR 'behavioral therapeutics' [All Fields] OR 'behavioral therapeutic' [All Fields] OR 'behavioral therapeutical' [All Fields] OR 'behavioral therapeutist' [All Fields] OR 'behavioral therapeutists' [All Fields] OR 'behavioral treatment' [All Fields] OR 'behavioral treatments' [All Fields] OR 'behaviour therapies' [All Fields] OR 'behaviour therapy' [All Fields] OR 'behaviour therapeutic' [All Fields] OR 'behaviour therapeutical' [All Fields] OR 'behaviour therapeutics' [All Fields] OR 'behaviour therapeutist' [All Fields] OR 'behaviour therapeutists' [All Fields] OR 'behaviour treatment' [All Fields] OR 'behaviour treatments' [All Fields] OR 'behaviours therapies' [All Fields] OR 'behaviours therapy' [All Fields] OR 'behaviours therapeutics' [All Fields] OR 'behaviours therapeutic' [All Fields] OR 'behaviours therapeutical' [All Fields] OR 'behaviours therapeutist' [All Fields] OR 'behaviours therapeutists' [All Fields] OR 'behaviours treatment' [All Fields] OR 'behaviours treatments' [All Fields] OR 'behavioural therapies' [All Fields] OR 'behavioural therapy' [All Fields] OR 'behavioural therapeutics' [All Fields] OR 'behavioural therapeutic' [All Fields] OR 'behavioural therapeutical' [All Fields] OR 'behavioural therapeutist' [All Fields] OR 'behavioural therapeutists' [All Fields] OR 'behavioural treatment' [All Fields] OR 'behavioural treatments' [All Fields] OR 'behavior activation' [All Fields] OR 'behaviors activation' [All Fields] OR 'behavioral activation' [All Fields] OR 'assertiveness training' [All Fields] OR 'cognition therapies' [All Fields] OR 'cognition therapie' [All Fields] OR 'cognition therapy' [All Fields] OR 'cognition therapeutical' [All Fields] OR 'cognition therapeutic' [All Fields] OR 'cognition therapeutics' [All Fields] OR 'cognition therapeutist' [All Fields] OR 'cognition therapeutists' [All Fields] OR 'cognition treatment' [All Fields] OR 'cognition treatments' [All Fields] OR 'cognitive therapies' [All Fields] OR 'cognitive therapy' [All Fields] OR 'cognitive therapeutic' [All Fields] OR 'cognitive therapeutics' [All Fields] OR 'cognitive therapeutical' [All Fields] OR 'cognitive therapeutist' [All Fields] OR 'cognitive therapeutists' [All Fields] OR 'cognitive treatment' [All Fields] OR 'cognitive treatments' [All Fields] OR 'cognitive restructuring' [All Fields] OR 'cognitive restructuring' [All Fields] OR problem-solving [All Fields] OR 'problem solving' [All Fields] OR 'acceptance commitment' [All Fields] OR 'acceptance and commitment' [All Fields] OR 'assertiveness training' [All Fields] OR MindfulnessOR 'solution-focused therapies' [All Fields] OR 'solution-focused therapy' [All Fields] OR 'solution-focused therapeutic' [All Fields] OR 'solution-focused therapeutics' [All Fields] OR 'solution-focused therapeutical' [All Fields] OR 'solution focused therapies' [All Fields] OR 'solution focused therapy' [All Fields] OR 'solution focused therapeutic' [All Fields] OR 'solution focused therapeutics' [All Fields] OR 'solution focused therapeutical' [All Fields] OR 'solution-focussed therapies' [All Fields] OR 'solution-focussed therapy' [All Fields] OR 'solution-focussed therapeutic' [All Fields] OR 'solution-focussed therapeutics' [All Fields] OR 'solution-focussed therapeutical'OR 'solution focussed therapies' [All Fields] OR 'solution focussed therapy' [All Fields] OR 'solution focussed therapeutic' [All Fields] OR 'solution focussed therapeutics' [All Fields] OR 'solution focussed therapeutical' [All Fields] OR DBT [All Fields] OR 'dialectical behavior therapy'

#2

Depression [Mesh Terms] OR 'Depressive Disorder' [Mesh Terms] OR 'major depression' [All Fields] OR 'major depressive disorder' [All Fields] OR depressions [All Fields] OR depressive [All Fields] OR dysthymi* [All Fields] OR 'affective disorder' [All Fields] OR 'affective disorders' [All Fields] OR 'mood disorder' [All Fields] OR 'mood disorders' [All Fields] OR depression* [All Fields] OR depressive* [All Fields] OR 'dysthymic disorder' [All Fields] OR dysthymia [All Fields] OR dysthymic [All Fields]

#3

Combine: #1 and #2

Limits: RCTs

Cochrane

#1

'behav* therap*' OR 'behavior therapy' OR 'behavior therapies' OR 'behavior therapeutic' OR 'behavior therapeutical' OR 'behavior therapeutics' OR 'behavior therapeutist' OR 'behavior therapeutists' OR 'behavior treatment' OR 'behavior treatments' OR 'behaviors therapies' OR 'behaviors therapy' OR 'behaviors therapeutics' OR 'behaviors therapeutic' OR 'behaviors therapeutical' OR 'behaviors therapeutist' OR 'behaviors therapeutists' OR 'behaviors treatment' OR 'behaviors treatments' OR 'behavioral therapies' OR 'behavioral therapy' OR 'behavioral therapeutics' OR 'behavioral therapeutic' OR 'behavioral therapeutical' OR 'behavioral therapeutist' OR 'behavioral therapeutists' OR 'behavioral treatment' OR 'behavioral treatments' OR 'behaviour therapies' OR 'behaviour therapy' OR 'behaviour therapeutic' OR 'behaviour therapeutical' OR 'behaviour therapeutics' OR 'behaviour therapeutist' OR 'behaviour therapeutists' OR 'behaviour treatment' OR 'behaviour treatments' OR 'behaviours therapies' OR 'behaviours therapy' OR 'behaviours therapeutics' OR 'behaviours therapeutic' OR 'behaviours therapeutical' OR 'behaviours therapeutist' OR 'behaviours therapeutists' OR 'behaviours treatment' OR 'behaviours treatments' OR 'behavioural therapies' OR 'behavioural therapy' OR 'behavioural therapeutics' OR 'behavioural therapeutic' OR 'behavioural therapeutical' OR 'behavioural therapeutist' OR 'behavioural therapeutists' OR 'behavioural treatment' OR 'behavioural treatments' OR 'behavior activation' OR 'behaviors activation' OR 'behavioral activation' OR 'assertiveness training' OR 'cognition therapies' OR 'cognition therapie' OR 'cognition therapy' OR 'cognition therapeutical' OR 'cognition therapeutic' OR 'cognition therapeutics' OR 'cognition therapeutist' OR 'cognition therapeutists' OR 'cognition treatment' OR 'cognition treatments' OR 'cognitive therapies' OR 'cognitive therapy' OR 'cognitive therapeutic' OR 'cognitive therapeutics' OR 'cognitive therapeutical' OR 'cognitive therapeutist' OR 'cognitive therapeutists' OR 'cognitive treatment' OR 'cognitive treatments' OR 'cognitive restructuring' OR 'cognitive restructuring' OR problem-solving OR 'problem solving' OR 'acceptance commitment' OR 'acceptance and commitment' OR 'assertiveness training' OR MindfulnessOR 'solution-focused therapies' OR 'solution-focused therapy' OR 'solution-focused therapeutic' OR 'solution-focused therapeutics' OR 'solution-focused therapeutical' OR 'solution focused therapies' OR 'solution focused therapy' OR 'solution focused therapeutic' OR 'solution focused therapeutics' OR 'solution focused therapeutical' OR 'solution-focussed therapies' OR 'solution-focussed therapy' OR 'solution-focussed therapeutic' OR 'solution-focussed therapeutics' OR 'solution-focussed therapeutical'OR 'solution focussed therapies' OR 'solution focussed therapy' OR 'solution focussed therapeutic' OR 'solution focussed therapeutics' OR 'solution focussed therapeutical' OR DBT OR 'dialectical behavior therapy' ' (Word variations have been searched)

#2

Depression OR 'Depressive Disorder' OR 'major depression' OR 'major depressive disorder' OR depressions OR depressive OR dysthymi* OR 'affective disorder' OR 'affective disorders' OR 'mood disorder' OR 'mood disorders' OR depression* OR depressive* OR 'dysthymic disorder' OR dysthymia OR dysthymic (Word variations have been searched)

#3

Combine: #1 and #2

#4

'Randomized controlled trial'

#5

Combine: #4 and #5

Limits: Trails

EmBase

#1

Depression OR 'Depressive Disorder' OR 'major depression' OR 'major depressive disorder' OR depressions OR depressive OR dysthymi* OR 'affective disorder' OR 'affective disorders' OR 'mood disorder' OR 'mood disorders' OR depression* OR depressive* OR 'dysthymic disorder' OR dysthymia OR dysthymic

#2

Psychotherapy OR psychotherap* OR psychotherapy OR psychotherapies OR psychotherapeutic OR psychotherapeutics OR psychotherapeutical OR cbt OR 'cognitive behavioural therapy' OR 'cognitive behavioural therapies' OR 'cognitive behavioral therapy' OR 'cognitive behavioral therapies' OR 'Cognitive Behav* therap*' OR 'behav* therap*' OR 'behavior therapy' OR 'behavior therapies' OR 'behavior therapeutic' OR 'behavior therapeutical' OR 'behavior therapeutics' OR 'behavior therapeutist' OR 'behavior therapeutists' OR 'behavior treatment' OR 'behavior treatments' OR 'behaviors therapies' OR 'behaviors therapy' OR 'behaviors therapeutics' OR 'behaviors therapeutic' OR 'behaviors therapeutical' OR 'behaviors therapeutist' OR 'behaviors therapeutists' OR 'behaviors treatment' OR 'behaviors treatments' OR 'behavioral therapies' OR 'behavioral therapy' OR 'behavioral therapeutics' OR 'behavioral therapeutic' OR 'behavioral therapeutical' OR 'behavioral therapeutist' OR 'behavioral therapeutists' OR 'behavioral treatment' OR 'behavioral treatments' OR 'behaviour therapies' OR 'behaviour therapy' OR 'behaviour therapeutic' OR 'behaviour therapeutical' OR 'behaviour therapeutics' OR 'behaviour therapeutist' OR 'behaviour therapeutists' OR 'behaviour treatment' OR 'behaviour treatments' OR 'behaviours therapies' OR 'behaviours therapy' OR 'behaviours therapeutics' OR 'behaviours therapeutic' OR 'behaviours therapeutical' OR 'behaviours therapeutist' OR 'behaviours therapeutists' OR 'behaviours treatment' OR 'behaviours treatments' OR 'behavioural therapies' OR 'behavioural therapy' OR 'behavioural therapeutics' OR 'behavioural therapeutic' OR 'behavioural therapeutical' OR 'behavioural therapeutist' OR 'behavioural therapeutists' OR 'behavioural treatment' OR 'behavioural treatments' OR 'behavior activation' OR 'behaviors activation' OR 'behavioral activation' OR 'assertiveness training' OR 'cognition therapies' OR 'cognition therapie' OR 'cognition therapy' OR 'cognition therapeutical' OR 'cognition therapeutic' OR 'cognition therapeutics' OR 'cognition therapeutist' OR 'cognition therapeutists' OR 'cognition treatment' OR 'cognition treatments' OR 'cognitive therapies' OR 'cognitive therapy' OR 'cognitive therapeutic' OR 'cognitive therapeutics' OR 'cognitive therapeutical' OR 'cognitive therapeutist' OR 'cognitive therapeutists' OR 'cognitive treatment' OR 'cognitive treatments' OR 'cognitive restructuring' OR 'cognitive restructuring' OR problem-solving OR 'problem solving' OR 'acceptance commitment' OR 'acceptance and commitment' OR 'assertiveness training' OR MindfulnessOR 'solution-focused therapies' OR 'solution-focused therapy' OR 'solution-focused therapeutic' OR 'solution-focused therapeutics' OR 'solution-focused therapeutical' OR 'solution focused therapies' OR 'solution focused therapy' OR 'solution focused therapeutic' OR 'solution focused therapeutics' OR 'solution focused therapeutical' OR 'solution-focussed therapies' OR 'solution-focussed therapy' OR 'solution-focussed therapeutic' OR 'solution-focussed therapeutics' OR 'solution-focussed therapeutical'OR 'solution focussed therapies' OR 'solution focussed therapy' OR 'solution focussed therapeutic' OR 'solution focussed therapeutics' OR 'solution focussed therapeutical' OR DBT OR 'dialectical behavior therapy'

#3

Combine: #1 and #2

Limitis: RCTs

Web of Science

#1

Depression OR 'Depressive Disorder' OR 'major depression' OR 'major depressive disorder' OR depressions OR depressive OR dysthymi* OR 'affective disorder' OR 'affective disorders' OR 'mood disorder' OR 'mood disorders' OR depression* OR depressive* OR 'dysthymic disorder' OR dysthymia OR dysthymic

#2

Psychotherapy OR psychotherap* OR psychotherapy OR psychotherapies OR psychotherapeutic OR psychotherapeutics OR psychotherapeutical OR cbt OR 'cognitive behavioural therapy' OR 'cognitive behavioural therapies' OR 'cognitive behavioral therapy' OR 'cognitive behavioral therapies' OR 'Cognitive Behav* therap*' OR 'behav* therap*' OR 'behavior therapy' OR 'behavior therapies' OR 'behavior therapeutic' OR 'behavior therapeutical' OR 'behavior therapeutics' OR 'behavior therapeutist' OR 'behavior therapeutists' OR 'behavior treatment' OR 'behavior treatments' OR 'behaviors therapies' OR 'behaviors therapy' OR 'behaviors therapeutics' OR 'behaviors therapeutic' OR 'behaviors therapeutical' OR 'behaviors therapeutist' OR 'behaviors therapeutists' OR 'behaviors treatment' OR 'behaviors treatments' OR 'behavioral therapies' OR 'behavioral therapy' OR 'behavioral therapeutics' OR 'behavioral therapeutic' OR 'behavioral therapeutical' OR 'behavioral therapeutist' OR 'behavioral therapeutists' OR 'behavioral treatment' OR 'behavioral treatments' OR 'behaviour therapies' OR 'behaviour therapy' OR 'behaviour therapeutic' OR 'behaviour therapeutical' OR 'behaviour therapeutics' OR 'behaviour therapeutist' OR 'behaviour therapeutists' OR 'behaviour treatment' OR 'behaviour treatments' OR 'behaviours therapies' OR 'behaviours therapy' OR 'behaviours therapeutics' OR 'behaviours therapeutic' OR 'behaviours therapeutical' OR 'behaviours therapeutist' OR 'behaviours therapeutists' OR 'behaviours treatment' OR 'behaviours treatments' OR 'behavioural therapies' OR 'behavioural therapy' OR 'behavioural therapeutics' OR 'behavioural therapeutic' OR 'behavioural therapeutical' OR 'behavioural therapeutist' OR 'behavioural therapeutists' OR 'behavioural treatment' OR 'behavioural treatments' OR 'behavior activation' OR 'behaviors activation' OR 'behavioral activation' OR 'assertiveness training' OR 'cognition therapies' OR 'cognition therapie' OR 'cognition therapy' OR 'cognition therapeutical' OR 'cognition therapeutic' OR 'cognition therapeutics' OR 'cognition therapeutist' OR 'cognition therapeutists' OR 'cognition treatment' OR 'cognition treatments' OR 'cognitive therapies' OR 'cognitive therapy' OR 'cognitive therapeutic' OR 'cognitive therapeutics' OR 'cognitive therapeutical' OR 'cognitive therapeutist' OR 'cognitive therapeutists' OR 'cognitive treatment' OR 'cognitive treatments' OR 'cognitive restructuring' OR 'cognitive restructuring' OR problem-solving OR 'problem solving' OR 'acceptance commitment' OR 'acceptance and commitment' OR 'assertiveness training' OR MindfulnessOR 'solution-focused therapies' OR 'solution-focused therapy' OR 'solution-focused therapeutic' OR 'solution-focused therapeutics' OR 'solution-focused therapeutical' OR 'solution focused therapies' OR 'solution focused therapy' OR 'solution focused therapeutic' OR 'solution focused therapeutics' OR 'solution focused therapeutical' OR 'solution-focussed therapies' OR 'solution-focussed therapy' OR 'solution-focussed therapeutic' OR 'solution-focussed therapeutics' OR 'solution-focussed therapeutical'OR 'solution focussed therapies' OR 'solution focussed therapy' OR 'solution focussed therapeutic' OR 'solution focussed therapeutics' OR 'solution focussed therapeutical' OR DBT OR 'dialectical behavior therapy'

#3

Combine: #1 and #2

#4

'Randomized controlled trial'

#5

Combine: #3 and #4 and Preprint Citation Index

PsycINFO

S1

Depression OR 'Depressive Disorder' OR 'major depression' OR 'major depressive disorder' OR depressions OR depressive OR dysthymi* OR 'affective disorder' OR 'affective disorders' OR 'mood disorder' OR 'mood disorders' OR depression* OR depressive* OR 'dysthymic disorder' OR dysthymia OR dysthymic

S2

Psychotherapy OR psychotherap* OR psychotherapy OR psychotherapies OR psychotherapeutic OR psychotherapeutics OR psychotherapeutical OR cbt OR 'cognitive behavioural therapy' OR 'cognitive behavioural therapies' OR 'cognitive behavioral therapy' OR 'cognitive behavioral therapies' OR 'Cognitive Behav* therap*' OR 'behav* therap*' OR 'behavior therapy' OR 'behavior therapies' OR 'behavior therapeutic' OR 'behavior therapeutical' OR 'behavior therapeutics' OR 'behavior therapeutist' OR 'behavior therapeutists' OR 'behavior treatment' OR 'behavior treatments' OR 'behaviors therapies' OR 'behaviors therapy' OR 'behaviors therapeutics' OR 'behaviors therapeutic' OR 'behaviors therapeutical' OR 'behaviors therapeutist' OR 'behaviors therapeutists' OR 'behaviors treatment' OR 'behaviors treatments' OR 'behavioral therapies' OR 'behavioral therapy' OR 'behavioral therapeutics' OR 'behavioral therapeutic' OR 'behavioral therapeutical' OR 'behavioral therapeutist' OR 'behavioral therapeutists' OR 'behavioral treatment' OR 'behavioral treatments' OR 'behaviour therapies' OR 'behaviour therapy' OR 'behaviour therapeutic' OR 'behaviour therapeutical' OR 'behaviour therapeutics' OR 'behaviour therapeutist' OR 'behaviour therapeutists' OR 'behaviour treatment' OR 'behaviour treatments' OR 'behaviours therapies' OR 'behaviours therapy' OR 'behaviours therapeutics' OR 'behaviours therapeutic' OR 'behaviours therapeutical' OR 'behaviours therapeutist' OR 'behaviours therapeutists' OR 'behaviours treatment' OR 'behaviours treatments' OR 'behavioural therapies' OR 'behavioural therapy' OR 'behavioural therapeutics' OR 'behavioural therapeutic' OR 'behavioural therapeutical' OR 'behavioural therapeutist' OR 'behavioural therapeutists' OR 'behavioural treatment' OR 'behavioural treatments' OR 'behavior activation' OR 'behaviors activation' OR 'behavioral activation' OR 'assertiveness training' OR 'cognition therapies' OR 'cognition therapie' OR 'cognition therapy' OR 'cognition therapeutical' OR 'cognition therapeutic' OR 'cognition therapeutics' OR 'cognition therapeutist' OR 'cognition therapeutists' OR 'cognition treatment' OR 'cognition treatments' OR 'cognitive therapies' OR 'cognitive therapy' OR 'cognitive therapeutic' OR 'cognitive therapeutics' OR 'cognitive therapeutical' OR 'cognitive therapeutist' OR 'cognitive therapeutists' OR 'cognitive treatment' OR 'cognitive treatments' OR 'cognitive restructuring' OR 'cognitive restructuring' OR problem-solving OR 'problem solving' OR 'acceptance commitment' OR 'acceptance and commitment' OR 'assertiveness training' OR MindfulnessOR 'solution-focused therapies' OR 'solution-focused therapy' OR 'solution-focused therapeutic' OR 'solution-focused therapeutics' OR 'solution-focused therapeutical' OR 'solution focused therapies' OR 'solution focused therapy' OR 'solution focused therapeutic' OR 'solution focused therapeutics' OR 'solution focused therapeutical' OR 'solution-focussed therapies' OR 'solution-focussed therapy' OR 'solution-focussed therapeutic' OR 'solution-focussed therapeutics' OR 'solution-focussed therapeutical'OR 'solution focussed therapies' OR 'solution focussed therapy' OR 'solution focussed therapeutic' OR 'solution focussed therapeutics' OR 'solution focussed therapeutical' OR DBT OR 'dialectical behavior therapy'

S3

DE (S1 AND S2)

Limits: Methodology is ME=(treatment outcome/clinical trial)
